# Study on electrochemical corrosion behavior of gray cast iron processed via laser cavitation peening

**DOI:** 10.1371/journal.pone.0353846

**Published:** 2026-07-16

**Authors:** Chunhui Luo, Jiayang Gu, Jiahao Yang, Yanfen Sun, Yuwen Ding, Aihua Xu, Xiaohu Sang

**Affiliations:** 1 School of Mechanical Engineering, Changzhou Vocational Institute of Mechatronic Technology, Changzhou, PR China; 2 School of Intelligent manufacturing, Jiangsu College of Engineering and Technology, Nantong, PR China; 3 School of Normal Education, Changzhou Institute of Technology, Changzhou, PR China; National Chung Cheng University College of Engineering, TAIWAN

## Abstract

Laser cavitation peening (LCP) is a novel material surface treatment method. In this study, we investigated the electrochemical corrosion performance of LCP-treated HT250 gray cast iron, with and without a coverage layer, under various laser energies, defocusing amounts, and immersion times. In the LCP process, a 0.04 mm aluminum coverage layer was employed. The influence of the coverage layer on LCP processing was analyzed. The results show that the presence of a coverage layer during the LCP treatment improves the specimen’s anti-corrosion ability, with this alignment corresponding with an increase in laser energy. In comparison with laser energy, the effect of a coverage layer on corrosion resistance at various defocusing amounts is not very significant, and at a smaller defocusing amount, the treated specimen presents higher corrosion resistance. The corrosion resistance of gray cast iron in the case with a coverage layer first increases with the increase in laser energy and then decreases. The presence of a coverage layer helps to avoid laser ablation and cavitation erosion.

## Introduction

Gray cast iron is widely applied in industries related to the use of hydraulic machinery, automobile brake drums, etc., due to its good casting properties, flexible machinability, and low cost [1–3]. Due to the fact that gray cast iron equipment usually works in a harsh liquid environment, the material’s serious corrosion problems need to be solved urgently [4]. Moreover, gray cast iron surfaces’ poor fatigue resistance and high brittleness limit their applications [5–6]. Many surface modification techniques, such as carburization [7–8], thermal spraying [9], plasma ion nitriding [10], and laser cladding [11], have been employed to improve the fatigue durability, corrosion resistance, and mechanical properties of gray cast iron. However, these methods have the drawbacks of creating an inadequate working environment, along with poor surface properties after processing.

Cavitation peening is an emerging surface modification method in which the impact of shock waves and water jets from the controlled collapse of cavitation bubbles near the solid boundary is used to induce compressive residual stress. Similar to shot peening and laser shock processing, the impact of cavitation peening causes the metal material to experience surface plastic deformation, ultimately improving the residual stress distribution and increasing the yield strength [12–13]. Soyama et al. [14–15] proved that this method can introduce compressive residual stress into remote areas of the material, and the treated material ultimately has lower surface roughness compared with other methods. Sonde et al. [16] built a cavitation peening model, encompassing a jet, bubble growth and collapse, a micro-jet, and residual stresses. Ijiri et al. [17] combined ultrasonic cavitation technology and water jet peening technology to improve the surface function of an Al–Cu alloy. It was found that multifunctional cavitation peening leads to high-temperature and high-pressure regimes, and the temperature and pressure in the cavitation bubbles depend on the standoff distance.

Laser cavitation is an important method for generating cavitation, which has the advantages of strong controllability and precise positioning. When the laser beam is focused on the liquid, the water will break down and form a plasma if the laser reaches the breakdown threshold of the liquid medium. Then, the plasma continues to absorb laser energy and expand to a cavitation bubble, which experiences the cyclical pulsation of “expansion–contraction”. Recently, there have been some efforts to investigate the mechanical effect and strengthening mechanism of laser-induced bubbles [18–20], and these efforts have proven that cavitation peening can introduce compressive residual stress and improve the micro-hardness of the material surface. However, few papers have focused on the electrochemical corrosion behavior of gray cast iron after laser cavitation peening (LCP) treatment, and the problems of laser ablation and cavitation erosion during the process of LCP also need to be resolved.

The aim of this study was to investigate the effect of coverage layer presence during LCP processing. In this study, a 0.04 mm aluminum coverage layer was employed in the LCP process. We investigated the electrochemical corrosion behavior of LCP-treated HT250 gray cast iron, with and without a coverage layer, at various laser energies, defocusing amounts, and immersion times. Herein, the effect of coverage layer presence is discussed.

## Experiments and methods

### Material and specimen pre-treatment

HT250 gray cast iron with dimensions of 20 mm (length) × 20 mm (width) × 8 mm (height) was selected as the material to be used in the experiment. Tables 1 and 2 show the chemical compositions and the mechanical properties of the HT250 specimens. Prior to LCP treatment, the top surfaces of all specimens were ground using various grades of SiC paper (from 180-grit to 2000-grit). Then, the specimens were immersed in C2H5OH and cleaned in an ultrasonic cleaner for 15 minutes, before finally being dried with a blower. Aluminum foil with a thickness of 0.1 mm was bonded to the polished surface of each specimen; this foil served as the coverage layer.

**Table 1:** Chemical composition of HT250 cast iron (wt %).

C	S	P	Mn	Si
3.16 - 3.30	0.09 - 0.13	0.12 - 0.17	0.84 - 1.04	1.79 - 1.93

**Table 2:** Mechanical properties of HT250 cast iron.

Properties	Tensile strength (MPa)	Hardness (HV)	Elongation (%)
Value	250	210 - 220	0.5

### LCP experiment device and procedure

The laser device we used is made up of a nanosecond laser (Nd:YAG dual-wavelength solid-state laser, Kinder Optoelectronics Technology Co., Ltd.), a laser energy meter (NIM-E1000, China Institute of Metrology), a reflector lens, a concave lens, a convex lens, and a 3D mobile platform (SIMUMEIK-840Di, SIMENS).

**Fig 1:**
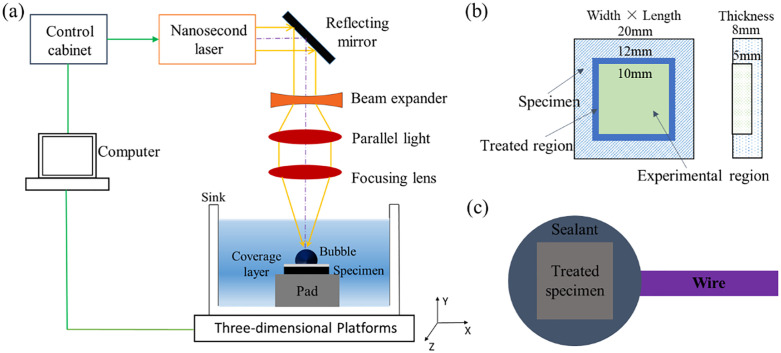
Experiment device and procedure (a) The schematic representation of LCP experimental system, (b) LCP treated specimen and (c) electrochemical corrosion specimen.

The experimental system used for LCP treatment is presented in Fig 1(a). Cavitation is induced by a Q-switched Nd: YAG laser with a 1064 nm wavelength and an 8 ns pulse duration. In order to concentrate the laser energy and obtain a single cavitation bubble, the laser beam was diverged using a concave lens and then converted into parallel light using a convex lens. Eventually, this laser beam was focused by the other convex lens and incident vertically into the liquid above the specimen; the diameter of the focused laser spot was 0.5 mm. The liquid depth was kept at a distance of 15 mm from the specimen surface. In the experiment, the laser energy meter was used to measure the laser energy, and the defocusing amount was adjusted through the 3D mobile platform. The laser energies used were 100 mJ, 200 mJ, 300 mJ, and 400 mJ, and the defocusing (the distance between the bubble center and the solid boundary) values were set to 0 mm, 1 mm, and 2 mm. The laser beam’s scanning path was set with a 50% overlap ratio, and the pulse frequency was set 1 Hz. The specimen region of LCP impact was 12 mm × 12 mm.

### Electrochemical corrosion experiments

In order to avoid the influence of specimen boundary effects on the experiments, a region of 10 mm × 10 mm × 5 mm (width × length × thickness) was cut from the middle of LCP-treated specimens (see Fig 1(b)), and then, the cut specimens were cleaned in ethanol, using an ultrasonic cleaner, for 10 minutes. The specimen used in the electrochemical corrosion experiment is depicted in Fig 1(c). The copper wire was welded on the opposite side of the specimen corrosion surface, and all the specimen surfaces were sealed with a sealant, except for the corrosion surface.

The electrochemical corrosion experiments were conducted on a CHI600E Electrochemical Workstation (Shanghai Chenhua Instrument Co., Ltd.), and a three-electrode system was employed. A platinum electrode, saturated calomel electrode, and the HT250 specimen acted as the auxiliary electrode, reference electrode, and working electrode, respectively. The corrosive electrolyte used was a 3.5% NaCl solution, which was prepared with NaCl of analytical purity and deionized water. The potentiodynamic polarization parameters and electrochemical impedance parameters of LCP-treated specimens were tested, under various laser energies and defocusing amounts, through the dynamic potential scanning method. Before that, however, the open-circuit potential was measured by immersing the specimens in a 3.5% NaCl solution for 15 minutes, and values reflecting the stable corrosion potential of the specimens were obtained. Moreover, the Tafel plot parameters and AC impedance parameters of the LCP-treated specimens under various immersion times (15 min, 30 min, 60 min, and 120 min) were also tested. In the test, the potential scan rate was set to 5 mV/s, and the scan potential varied from −2 V to −0 V. The test frequency of the electrochemical impedance spectrum ranged from 10−2–104 Hz, and the voltage amplitude was 5 × 10−3 V. All the experiments were performed at ambient temperature. Two specimens were used for each repeat experiment, and the average values of the parameters were obtained. In addition, ZSimpWin software was used to fit the electrochemical impedance spectroscopy curve.

### Measurements of residual stress and surface roughness

**Fig 2:**
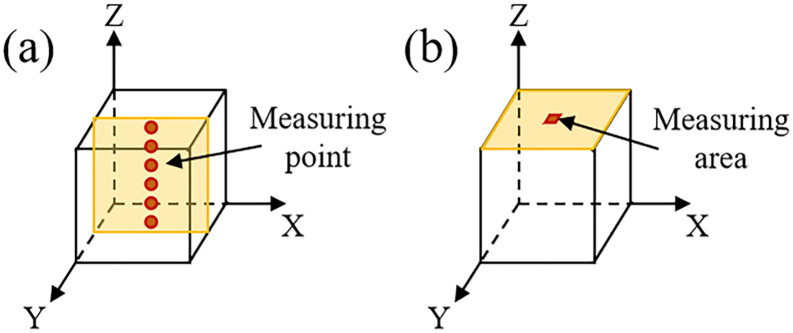
Experimental set up (a) Measuring points of residual stress, (b) Measuring area of surface roughness.

The residual stress was measured using an X-350A residual stress tester with X-ray diffraction. The diffraction crystal plane was (2 1 1), the scanning angle ranged from 150° to 162°, and the feed angle of the ladder scanning was 0.1°/s. The measuring points of residual stress (11 points in total) are shown in Fig 2(a), and the specimens needed to be treated through a controlled layer-based removal method during the measurements. The surface roughness of all cast iron specimens was measured using an ultra-high-resolution true color confocal microscope (Axio CSM 700), measuring the Sa of the center area (300 μm × 300 μm) on the specimen surface, as shown in Fig 2(b).

## Results and discussion

### Electrochemical corrosion performance at various laser energies and defocusing amounts

Laser energy and defocusing amount are two key parameters in LCP. In this section, the effects of coverage layer presence on electrochemical corrosion performance under various laser energies and laser energies are discussed.

#### Potentiodynamic polarization curve.

**Fig 3:**
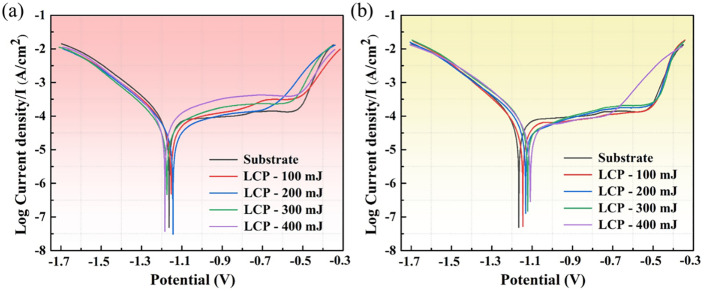
The potentiodynamic polarization curves of HT250 specimens under various laser energies at the case of (a) no coverage layer and (b) coverage layer.

Fig 3 shows the potentiodynamic polarization curves of the substrate and LCP-treated specimens at various laser energies in a 3.5% NaCl solution. The corrosion potentials and corrosion current densities of various LCP-treated specimens were obtained through the Tafel extrapolation method; the values derived are listed in Table 3. At 100 mJ and 200 mJ laser energy, it can be observed that the polarization curves of the LCP-treated specimens all shift positively both with and without a coverage layer, and the corrosion current density also decreases. This indicates that the corrosion rate of the LCP-treated specimens decreases and that the corrosion resistance improves compared to the substrate. However, the polarization curves’ shifts exhibit obvious differences at 300 mJ and 400 mJ laser energy. As shown in Fig 3(a), the polarization curves shift negatively as the laser energy rises to 300 mJ and 400 mJ, and the corrosion potentials are −1.174 V and −1.182 V, exhibiting a decrease compared to the substrate. However, as is also shown in Fig 3(b), the polarization curves shift positively when there is a coverage layer, and the corrosion potentials are −1.128 V and −1.112 V (300 mJ and 400 mJ). The presence of a coverage layer during the LCP treatment improves the specimen’s anti-corrosion ability with the increase in laser energy.

**Table 3:** Corrosion potentials and corrosion current densities of the specimens from the obtained from the potentiodynamic polarization curves.

Specimen	Corrosion potential (V)	Corrosion current density (μA/cm^2^)
Substrate	−1.164 ± 0.027	91.55 ± 2.9
LCP - 100mJ - 1 mm	−1.152 ± 0.019	85.73 ± 3.1
LCP - 200mJ - 1 mm	−1.143 ± 0.021	79.13 ± 1.6
LCP - 300mJ - 1 mm	−1.174 ± 0.023	91.66 ± 2.2
LCP - 400mJ - 1 mm	−1.182 ± 0.018	102.36 ± 2.7
LCP - 200mJ - 0 mm	−1.185 ± 0.016	113.24 ± 2.8
LCP - 200mJ - 2 mm	−1.170 ± 0.024	81.43 ± 3.4
*LCP - 100mJ - 1 mm	−1.147 ± 0.019	76.59 ± 3.7
*LCP - 200mJ - 1 mm	−1.135 ± 0.018	69.42 ± 1.8
*LCP - 300mJ - 1 mm	−1.128 ± 0.021	74.13 ± 3.2
*LCP - 400mJ - 1 mm	−1.112 ± 0.024	63.56 ± 3.1
*LCP - 200mJ - 0 mm	−1.158 ± 0.018	98.16 ± 3.3
*LCP - 200mJ - 2 mm	−1.137 ± 0.019	86.53 ± 2.6

* With coverage layer.

**Fig 4:**
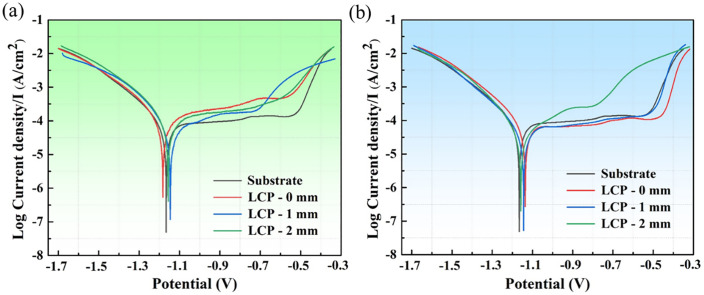
The potentiodynamic polarization curves of HT250 specimens under various defocusing amounts at the case of (a) no coverage layer and (b) coverage layer.

The potentiodynamic polarization curves of the substrate and LCP-treated specimens at various defocusing amounts are presented in Fig 4. It can be seen from Fig 4(a) that the polarization curve of the LCP-treated specimens shifts positively at 1 mm and 2 mm defocusing amounts, while it shifts towards negative values at 0 mm. The corrosion current density is consistent with this trend, and the corrosion current density at 0 mm reaches 113.24 μA/cm2. The polarization curves have a passivation tendency, and for the specimens without a coverage layer, the pitting phenomenon is not obvious. As shown in Fig 4(b), the polarization curve for LCP-treated specimens with a coverage layer always shifts positively, and the corrosion potential increases with a decrease in the defocusing amount. The values of corrosion potential at three different defocusing amounts are −1.158 V, −1.135 V, and −1.137 V. In comparison with laser energy, the effect of coverage layer presence on corrosion resistance at various defocusing amounts is not very significant.

#### Electrochemical impedance spectroscopy.

**Fig 5:**
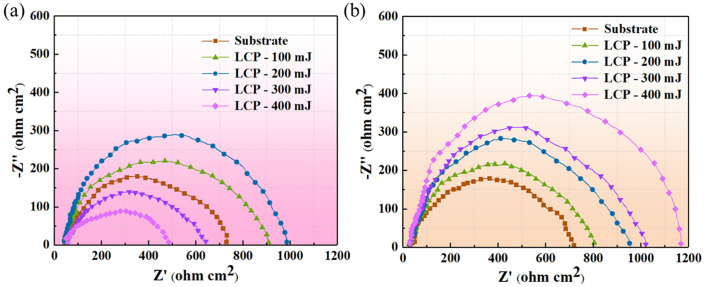
The electrochemical impedance spectroscopy of HT250 specimens under various laser energies at the case of (a) no coverage layer and (b) coverage layer.

Electrochemical impedance spectroscopy (EIS) was employed to evaluate the electrochemical corrosion performance of the HT250 specimens, helping to determine parameters including resistance, capacitance, and so on [21]. The EIS results for the substrate and LCP-treated specimens, including those with and without a coverage layer, under various laser energies are shown in Fig 5. The charge transfer appears at the interfaces between one specimen surface and the electrical double-layer capacitor in the NaCl electrolyte solution, so the EIS results present semicircular-shaped high-frequency capacitive arcs. It can be seen from Fig 5(a) that the capacitance arc radius of the specimen without a coverage layer is LCP – 200 mJ > LCP – 100 mJ > substrate > LCP – 300 mJ > LCP – 400 mJ. As we know, the capacitance arc radius has a positive correlation with the specimen impedance, so the corrosion rate of the specimen is lower and the anti-corrosion is better when the capacitance arc radius is larger [22]. Therefore, the corrosion resistance of specimens without a coverage layer is improved at 100 mJ and 200 mJ laser energy, while it is weaker at 300 mJ and 400 mJ. As shown in Fig 5(b), there is a larger capacitive arc radius for the LCP-treated specimen at a higher laser energy. Similarly to the results for corrosion potential, the impedance value of the LCP-treated specimens with a coverage layer increases with the increase in laser energy.

**Fig 6:**
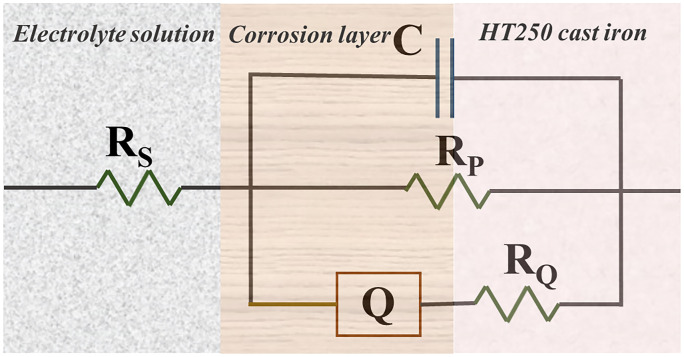
The equivalent circuit model of electrochemical impedance spectroscopy.

The specimens’ EIS results were fitted through the use of ZSimpWin software so as to better investigate the electrochemical corrosion behavior. Fig 6 shows the established equivalent circuit model R(CR(QR)), and the fitting parameter values are listed in Table 4. The parameters in the equivalent circuit include the electrolyte solution RS, capacitance C, capacitance resistance RQ, charge transfer resistance RP, and the non-ideal electric double-layer capacitance Q in the electrolyte solution. Q is defined by two parameters, Y and n. Q is purely capacitive when n = 1. As can be seen in the table, the charge transfer resistances RP of the LCP-treated specimens with a coverage layer are all higher than that of the substrate, and the maximum RP value for specimens without a coverage layer appears at an LCP energy of 200 mJ.

**Table 4:** The fitted parameters of specimens obtained from the electrochemical impedance spectroscopy curves.

Specimen	Rs (Ω· cm^2^)	C (F· cm^-2^)	R_P_ (Ω· cm^2^)	Q_Y_ (Ω^-1^· cm^-2^· S^n^)	Q_n_ (0 < n < 1)	R_Q_ (Ω· cm^2^)	χ^2^ (× 10^−4^)
Substrate	8.25 ± 0.073	(6.635 ± 0.689)×10^−5^	518.3 ± 6.1	(6.535 ± 0.112)×10^−4^	0.892 ± 0.01	5.78 ± 0.96	3.76
LCP - 100mJ - 1 mm	6.49 ± 0.057	(5.392 ± 0.586)×10^−5^	579.6 ± 5.2	(9.516 ± 0.205)×10^−4^	0.916 ± 0.01	6.36 ± 0.75	4.28
LCP - 200mJ - 1 mm	8.71 ± 0.066	(6.103 ± 0.625)×10^−5^	613.6 ± 5.7	(5.236 ± 0.093)×10^−4^	0.836 ± 0.02	3.65 ± 0.68	3.91
LCP - 300mJ - 1 mm	5.72 ± 0.083	(4.955 ± 0.524)×10^−5^	527.3 ± 7.8	(11.220 ± 0.217)×10^−4^	0.878 ± 0.01	5.14 ± 0.73	4.55
LCP - 400mJ - 1 mm	7.63 ± 0.061	(7.678 ± 0.817)×10^−5^	489.3 ± 6.5	(6.897 ± 0.175)×10^−4^	0.908 ± 0.02	3.58 ± 0.61	3.14
LCP - 200mJ - 0 mm	7.12 ± 0.078	(9.653 ± 0.986)×10^−5^	474.5 ± 10.6	(7.633 ± 0.143)×10^−4^	0.885 ± 0.01	5.66 ± 0.92	4.83
LCP - 200mJ - 2 mm	6.39 ± 0.052	(5.658 ± 0.588)×10^−5^	551.7 ± 9.3	(10.810 ± 0.128)×10^−4^	0.887 ± 0.01	4.32 ± 0.80	3.47
*LCP - 100mJ - 1 mm	5.47 ± 0.059	(4.737 ± 0.513)×10^−5^	568.6 ± 6.6	(8.586 ± 0.183)×10^−4^	0.922 ± 0.01	2.97 ± 0.45	6.72
*LCP - 200mJ - 1 mm	9.16 ± 0.082	(8.568 ± 0.897)×10^−5^	626.3 ± 5.9	(6.281 ± 0.096)×10^−4^	0.912 ± 0.01	5.69 ± 0.85	3.69
*LCP - 300mJ - 1 mm	7.28 ± 0.039	(6.316 ± 0.683)×10^−5^	798.8 ± 4.7	(7.592 ± 0.106)×10^−4^	0.865 ± 0.02	2.97 ± 0.45	4.97
*LCP - 400mJ - 1 mm	6.34 ± 0.026	(7.426 ± 0.802)×10^−5^	935.8 ± 8.1	(12.670 ± 0.129)×10^−4^	0.876 ± 0.02	4.76 ± 0.78	3.25
*LCP - 200mJ - 0 mm	5.98 ± 0.052	(5.886 ± 0.626)×10^−5^	825.7 ± 9.2	(5.965 ± 0.153)×10^−4^	0.861 ± 0.01	3.64 ± 0.71	3.27
*LCP - 200mJ - 2 mm	6.55 ± 0.065	(6.395 ± 0.452)×10^−5^	676.6 ± 8.5	(8.635 ± 0.137)×10^−4^	0.889 ± 0.01	5.35 ± 0.83	6.02

* With coverage layer.

**Fig 7:**
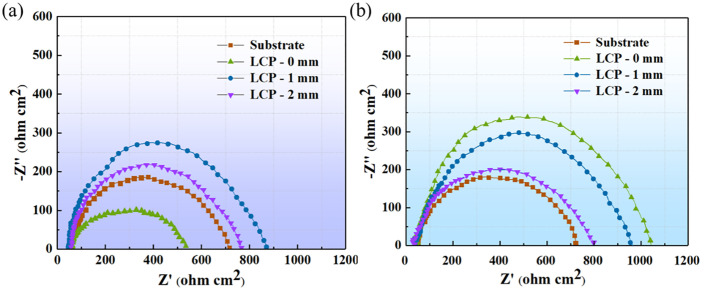
The electrochemical impedance spectroscopy of HT250 specimens under various defocusing amounts at the case of (a) no coverage layer and (b) coverage layer.

Fig 7 shows the electrochemical impedance spectroscopy results of the substrate and LCP-treated specimens for the two experimental cases (with and without coverage layer) under various defocusing amounts. As shown in Fig 7(a), the maximum capacitance arc radius of the specimen appears at 1 mm defocusing amount, and the value of RP is 613.6 Ω. The arc radius for the no-coverage layer case is LCP – 1 mm > LCP – 2 mm > substrate > LCP – 0 mm, so the specimen’s anti-corrosion ability is weakened at a defocusing amount of 0 mm. It can be observed from Fig 7(b) that the capacitance arc radius of the LCP-treated specimens is larger than that of the substrate, and the arc radius increases with a decrease in defocusing amount. Therefore, the specimen’s corrosion resistance improves at all defocusing amounts after the treatment with a coverage layer, and LCP treatment at a smaller defocusing amount leads to higher corrosion resistance.

#### Corrosion morphology.

**Fig 8:**
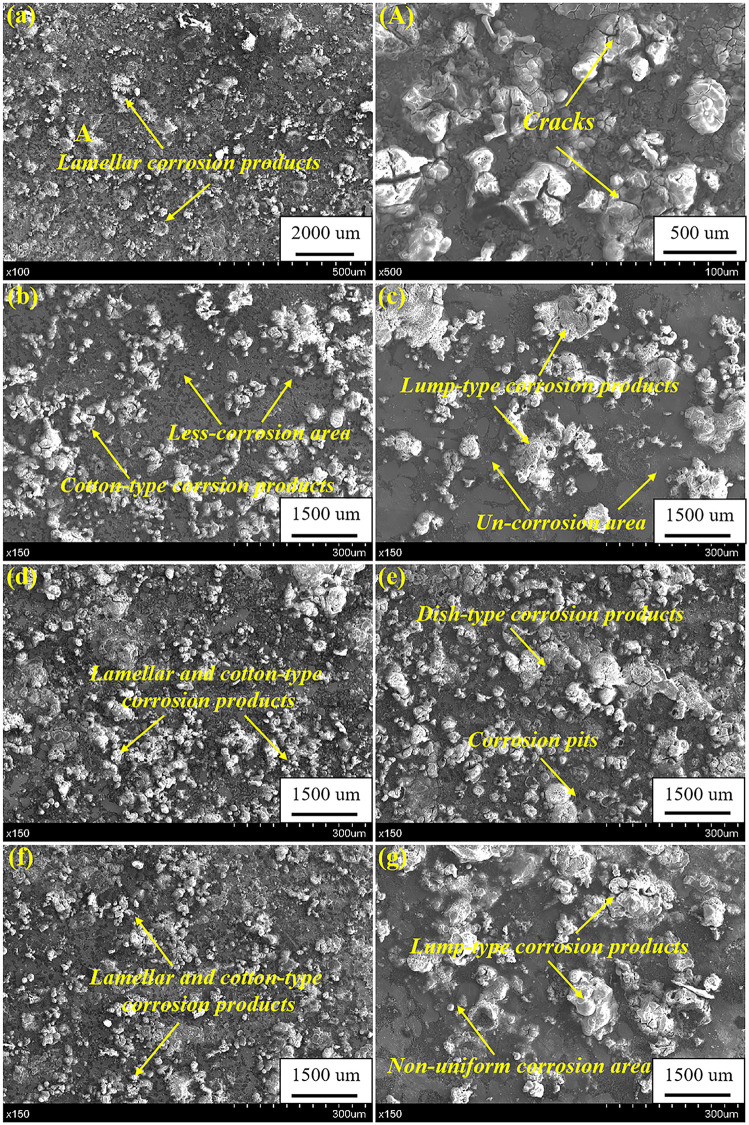
SEM micrographs of various HT250 specimens at the case of no coverage layer after electrochemical corrosion: (a) substrate, (b) LCP – 100 mJ - 1 mm, (c) LCP – 200 mJ - 1 mm, (d) LCP – 300 mJ - 1 mm, (e) LCP – 400 mJ - 1 mm, (f) LCP – 200 mJ - 0 mm and (g) LCP – 200 mJ - 2 mm.

The surface morphologies of the substrate and LCP-treated specimens after electrochemical corrosion for the no-coverage layer case are presented in Fig 8. It can be seen from Fig 8(a) and (A) that the substrate’s anti-corrosion ability is relatively poor, and after corrosion, the surface is almost completely covered by the corrosion products. The corrosion products present a lamellar shape, and some cracks appear on the upper side of the white corrosion layer. As shown in Fig 8(b) and 8(c), many cotton-type and lump-type corrosion products are generated on the specimen surface, but the specimen surface appears to have an area with less corrosion and an uncorroded area, which indicates that the extent of electrochemical corrosion decreases significantly after LCP treatment at – 100 mJ - 1 mm and LCP – 200 mJ - 1 mm. The corrosion resistance of LCP (LCP – 300 mJ - 1 mm and LCP – 400 mJ - 1 mm)-treated specimens is lower than that of the substrate, and the specimen surface was severely corroded, as shown in Fig 8(d) and 8(e). After the electrochemical corrosion, the surfaces of the specimens were covered with thick lamellar and dish-type corrosion products, and corrosion pits began to form. As shown in Fig 8(f), the anti-corrosion of the LCP-treated specimen (treated at – 200 mJ - 0 mm) is still lower than that of the substrate. The specimen surface has many lamellar and cotton-type corrosion products, and the distribution is uneven. The corrosion products on the surface of the LCP (LCP – 200 mJ - 2 mm)-treated specimen present a lump-like shape, and the distribution is also non-uniform (see Fig 8(g)).

**Fig 9:**
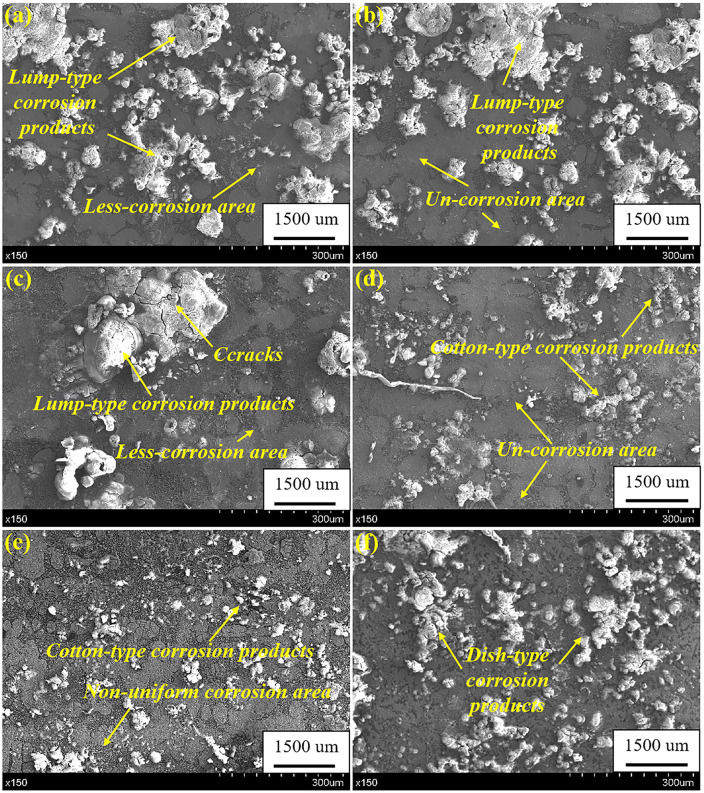
SEM micrographs of various HT250 specimens at the case of coverage layer after electrochemical corrosion: (a) LCP – 100 mJ - 1 mm, (b) LCP – 200 mJ - 1 mm, (c) LCP – 300 mJ - 1 mm, (d) LCP – 400 mJ - 1 mm, (e) LCP – 200 mJ - 0 mm and (f) LCP – 200 mJ - 2 mm.

Fig 9 shows the surface morphologies of the LCP-treated specimens with a coverage layer after electrochemical corrosion. The corrosion resistance of these specimens improved significantly and increased with the increase in laser energy, and the corrosion morphology is consistent with this trend. As can be seen in the referenced figure, the extent of electrochemical corrosion on the LCP-treated specimens with a coverage layer is much lower than that without a coverage layer. In Fig 9(a) and 9(b), the surfaces of the LCP – 100 mJ - 1 mm and LCP – 200 mJ - 1 mm specimens are covered with some big lump-type corrosion products, but there are also many less corroded and uncorroded areas. As shown in Fig 9(c), the lump-type corrosion products on the LCP – 300 mJ - 1 mm specimen surface become big and concentrated, and cracks appear on the corrosion products, but the range of the lower-corrosion area is enlarged. As the laser energy increases to 400 mJ (see Fig 9(d)), the corrosion products present a cotton-like shape, and the range of the uncorroded area is further enlarged. It can be observed from Fig 9(e) that the cotton-type corrosion products are smaller and sparse on the surface of the LCP – 200 mJ - 0 mm specimen, and the distribution of corrosion is non-uniform. The corrosion of the LCP – 200 mJ - 2 mm specimen (see Fig 9(f)) seems relatively serious compared to the other specimens, and many dish-type corrosion products cover the surface.

### Electrochemical corrosion performance as a function of immersion time

**Fig 10:**
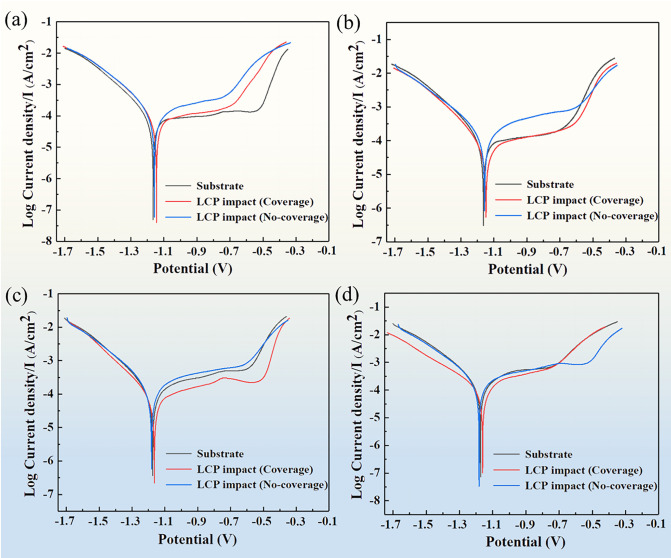
The potentiodynamic polarization curves of HT250 specimens at the case of no coverage layer and coverage layer under various immersion of (a) 15 min (b) 30 min, (c) 60 min and (d) 120 min.

The effects of the coverage layer on electrochemical corrosion performance were investigated under various immersion times (15 min, 30 min, 60 min, and 120 min). All the experiments were performed at 200 mJ laser energy and with a 1 mm defocusing amount. Fig 10 shows the potentiodynamic polarization curves of the substrate and LCP-treated specimens for the two experimental cases (with and without a coverage layer) under various immersion times in a 3.5% NaCl solution. The corrosion potentials and the corrosion current densities of various specimens are listed in Table 5. As shown in Fig 10(a), compared with the substrate and under 15 min immersion, the corrosion potential of the LCP-treated specimens increases, while the corrosion current density decreases. For the two experimental cases, the anti-corrosion ability of all of the LCP-treated specimens was improved. It can be observed from Fig 10(b) that the corrosion potentials all increase under 30 min immersion, but the positive shift of the polarization curve is not obvious in the no-coverage layer case. Moreover, the current density decreases in the no-coverage layer case, before increasing to 103.44 μA/cm2, which indicates a correlation with the rise in corrosion rate. As the immersion time increases to 60 min and 120 min, the polarization curves of all specimens shift negatively, as shown in Fig 10(c) and 10(d). The specimens’ corrosion resistance decreases significantly, and the anti-corrosion ability of the LCP-treated specimens in the no-coverage layer case is weaker than that of the substrate. In particular, the corrosion potentials decrease to – 1.178 V, −1.183 V, and −1.172 V after immersion for 120 min.

**Table 5:** Corrosion potentials and corrosion current densities of the specimens from the obtained from the potentiodynamic polarization curves.

Immersion time (min)	Specimen	Corrosion potential (V)	Corrosion current density (μA/cm^2^)
15	Substrate	−1.164 ± 0.027	91.55 ± 2.9
15	LCP - 200mJ - 1 mm	−1.143 ± 0.021	79.13 ± 1.6
15	*LCP - 200mJ - 1 mm	−1.135 ± 0.018	69.42 ± 1.8
30	Substrate	−1.166 ± 0.021	98.86 ± 2.2
30	LCP - 200mJ - 1 mm	−1.162 ± 0.024	103.44 ± 3.7
30	*LCP - 200mJ - 1 mm	−1.153 ± 0.019	85.32 ± 2.8
60	Substrate	−1.175 ± 0.031	108.78 ± 2.6
60	LCP - 200mJ - 1 mm	−1.177 ± 0.018	93.79 ± 2.7
60	*LCP - 200mJ - 1 mm	−1.169 ± 0.022	88.65 ± 3.1
120	Substrate	−1.178 ± 0.024	96.23 ± 2.7
120	LCP - 200mJ - 1 mm	−1.183 ± 0.026	108.67 ± 3.3
120	*LCP - 200mJ - 1 mm	−1.172 ± 0.019	95.73 ± 2.8

* With coverage layer.

**Fig 11:**
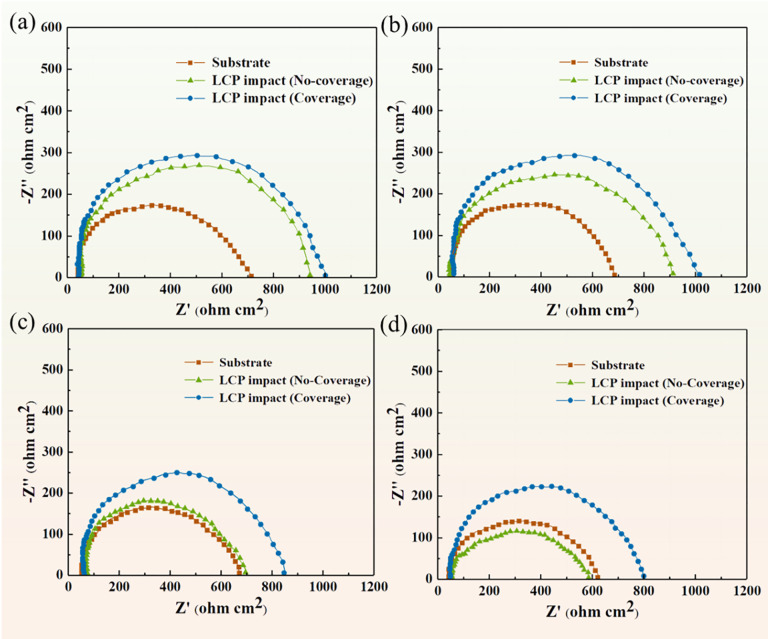
The electrochemical impedance spectroscopy of HT250 specimens at the case of no coverage layer and coverage layer under various immersion of (a) 15 min (b) 30 min, (c) 60 min and (d) 120 min.

Fig 11 shows the EIS results of the substrate and LCP-treated specimens for the two cases under various immersion times, and the fitting parameters are listed in Table 6. As can be seen in the figure, the capacitance arc radius of the LCP specimens with a coverage layer is always bigger for each immersion time, and all the arc radii decrease as the immersion time increases. At 15 min, 30 min, and 60 min immersion time (see Fig 11a–11c), the arc radii of the LCP-treated specimens are all larger than that of substrate, but the arc radius for the no-coverage layer case is almost the same as the substrate under 60 min immersion time. As shown in Fig 11(d), the arc radius of the LCP-treated specimen for the no-coverage layer case is smaller than that of the substrate after 120 min immersion, but the arc radius for the coverage layer case is still larger. The change in charge transfer resistance RP is consistent with this trend. LCP – 200 mJ - 1 mm improves the specimens’ corrosion resistance for both the no-coverage layer case and the coverage layer case, but the anti-corrosion ability decreases with increasing immersion time.

**Table 6:** The fitted parameters of specimens obtained from the electrochemical impedance spectroscopy curves.

Immersion time (min)	Specimen	Rs (Ω· cm^2^)	C (F· cm^-2^)	R_P_ (Ω· cm^2^)	Q_Y_ (Ω^-1^· cm^-2^· S^n^)	Q_n_ (0 < n < 1)	R_Q_ (Ω· cm^2^)
15	Substrate	8.25 ± 0.073	(6.265 ± 0.693)×10^−5^	518.3 ± 6.1	(5.633 ± 0.103)×10^−4^	0.892 ± 0.01	5.78 ± 0.96
15	LCP - 200mJ - 1 mm	6.49 ± 0.057	(5.364 ± 0.605)×10^−5^	613.6 ± 5.7	(6.526 ± 0.127)×10^−4^	0.836 ± 0.02	6.36 ± 0.75
15	*LCP - 200mJ - 1 mm	8.71 ± 0.066	(7.213 ± 0.788)×10^−5^	626.3 ± 5.9	(8.278 ± 0.165)×10^−4^	0.912 ± 0.01	3.65 ± 0.68
30	Substrate	6.58 ± 0.072	(5.935 ± 0.663)×10^−5^	486.3 ± 6.5	(8.521 ± 0.171)×10^−4^	0.863 ± 0.01	3.79 ± 0.76
30	LCP - 200mJ - 1 mm	7.24 ± 0.063	(4.773 ± 0.561)×10^−5^	509.5 ± 5.4	(7.652 ± 0.146)×10^−4^	0.922 ± 0.01	4.64 ± 0.71
30	*LCP - 200mJ - 1 mm	8.33 ± 0.086	(6.653 ± 0.724)×10^−5^	532.2 ± 9.3	(10.873 ± 0.215)×10^−4^	0.896 ± 0.01	6.35 ± 0.85
60	Substrate	5.39 ± 0.061	(8.857 ± 0.929)×10^−5^	457.1 ± 8.3	(12.117 ± 0.246)×10^−4^	0.871 ± 0.02	3.32 ± 0.62
60	LCP - 200mJ - 1 mm	5.73 ± 0.054	(6.637 ± 0.717)×10^−5^	463.4 ± 6.1	(8.596 ± 0.178)×10^−4^	0.885 ± 0.01	4.97 ± 0.82
60	*LCP - 200mJ - 1 mm	8.26 ± 0.076	(7.342 ± 0.798)×10^−5^	514.3 ± 6.7	(5.781 ± 0.145)×10^−4^	0.903 ± 0.01	3.68 ± 0.76
120	Substrate	7.85 ± 0.062	(5.362 ± 0.613)×10^−5^	379.6 ± 4.7	(7.692 ± 0.153)×10^−4^	0.914 ± 0.02	2.67 ± 0.43
120	LCP - 200mJ - 1 mm	9.36 ± 0.088	(5.836 ± 0.662)×10^−5^	435.2 ± 6.2	(11.371 ± 0.223)×10^−4^	0.867 ± 0.02	3.36 ± 0.57
120	*LCP - 200mJ - 1 mm	8.09 ± 0.078	(6.913 ± 0.753)×10^−5^	477.8 ± 8.9	(7.765 ± 0.156)×10^−4^	0.891 ± 0.01	4.11 ± 0.65

* With coverage layer.

**Fig 12:**
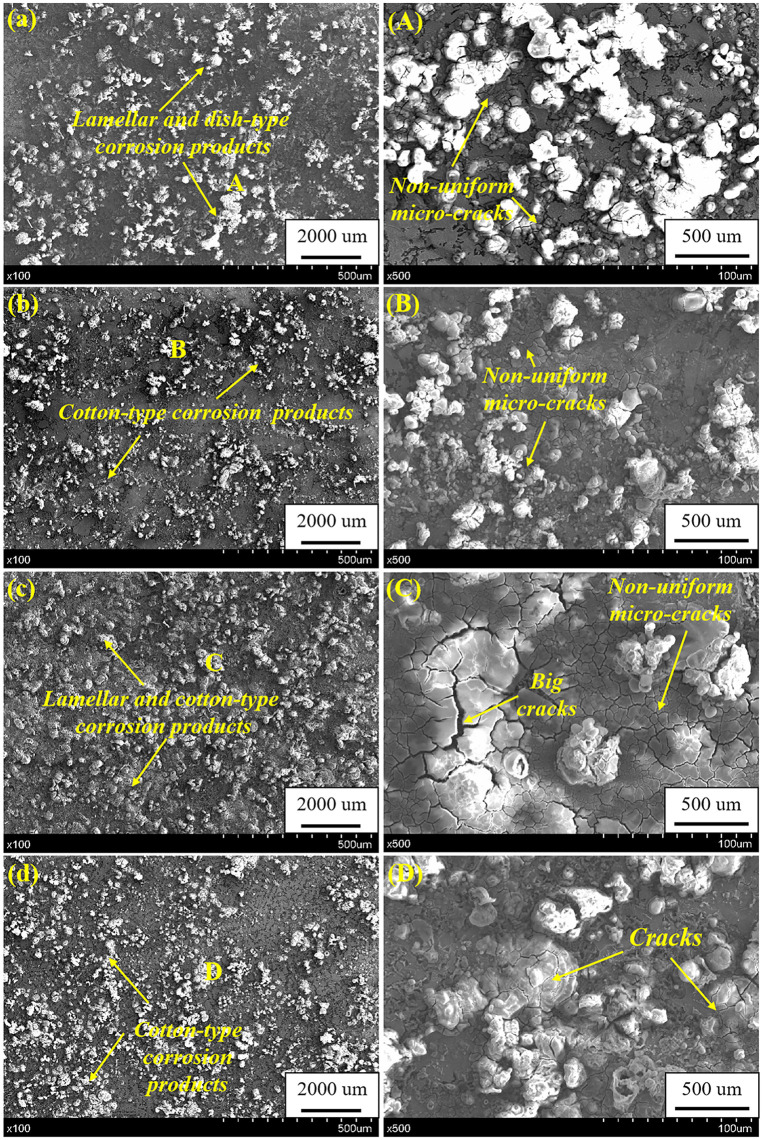
SEM micrographs of LCP – 200 mJ - 1 mm treated HT250 specimens under various immersion time after electrochemical corrosion: (a) 60 min, (b) 60 min + coverage layer, (c) 120 min, (d) 120 min + coverage layer.

Fig 12 presents the surface morphologies of the substrate and LCP-treated specimens for the two experimental cases after electrochemical corrosion for 60 min and 120 min immersion time. It can be observed that the specimen surfaces were seriously corroded and covered with a large amount of corrosion products. In Fig 12(a) and (A), many white lamellar and dish-type corrosion products are on the specimen surface, and some non-uniform micro-cracks can be found when the corrosion products are amplified 500 times. For the coverage layer case, the region of corrosion is uneven under 60 min immersion time, and the cotton-type corrosion products on the specimen surface are smaller, as shown in Fig 12(b). The micro-cracks are also non-uniform and smaller than those for the no-coverage layer case (see Fig 12(B)). As seen in Fig 12(c), the extent of electrochemical corrosion after 120 min immersion for the no-coverage layer case is most serious. Lots of lamellar and cotton-type corrosion products appear on the specimen, and the corrosion products covering the specimen surface are dense. There is a large number of non-uniform micro-cracks on the corrosion products, and there are some big cracks in the local region (see Fig 12(C)). As shown in Fig 12(d) and (D), the corrosion products for the coverage layer case are smaller and seemingly present as cotton-type products, and the cracks decrease significantly.

**Fig 13:**
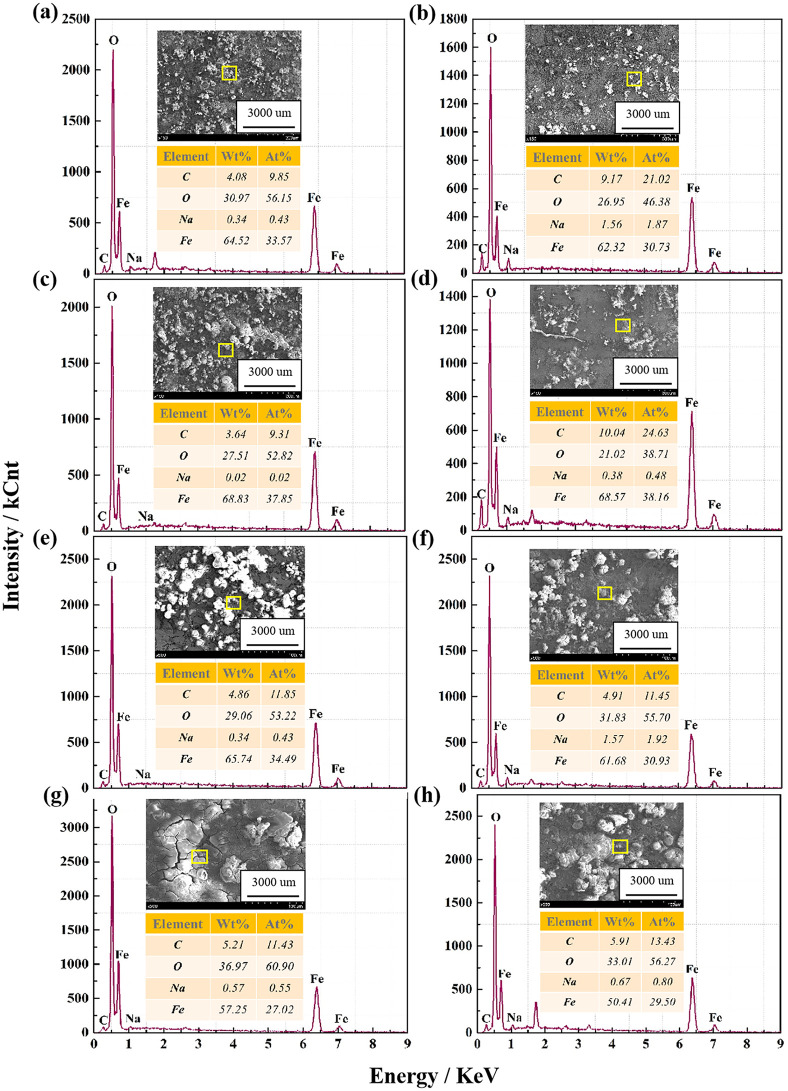
The energy spectrum of various HT250 specimens under various immersion time after electrochemical corrosion: (a) LCP – 200 mJ - 0 mm + 15 min, (b) LCP – 200 mJ - 0 mm (coverage layer) + 15 min, (c) LCP – 400 mJ - 1 mm + 15 min, (d) LCP – 400 mJ - 1 mm (coverage layer) + 15 min, (e) LCP – 200 mJ - 1 mm + 60 min, (f) LCP – 200 mJ - 1 mm (coverage layer) + 60 min, (g) LCP – 200 mJ - 1 mm + 120 min (h) LCP – 200 mJ - 1 mm (coverage layer) + 120 min.

EDS was employed to detect the components of the corrosion products, and the results are shown in Fig 13. It can be seen that the corrosion products of the specimen include Fe, O, C, and Cl elements. The Fe and C elements are the original elements of the HT250 specimen, and the presence of the Cl element may be explained by the invasion of Cl ions on the specimen surface when the specimen is corroded in the NaCl solution. The content of Fe and O elements in each specimen is very high, which indicates that a large amount of Fe element was oxidized and that the generation of compounds took place during the process of electrochemical corrosion. The O element content is higher in the LCP-treated specimens without a coverage layer and subjected to a long immersion time, and a higher O element content means the extent of corrosion is higher.

### Residual stress and surface roughness

**Fig 14:**
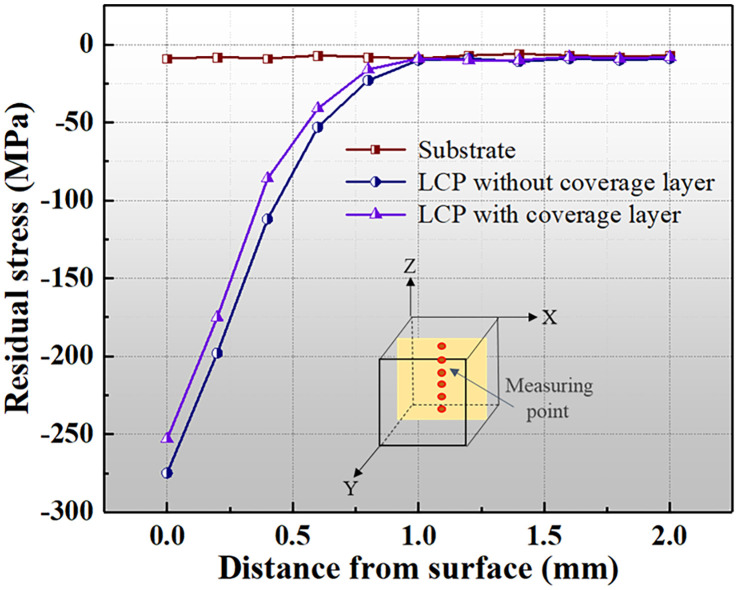
The residual stress distribution along the depth direction of substrate and LCP treated HT250 specimens at the case of coverage and no coverage layer.

Fig 14 shows the residual stress profiles of the substrate and various HT250 specimens after LCP – 200 mJ - 1 mm treatment along the depth direction. The residual stress of the untreated specimen is – 9 MPa, and the influence on the experiment can be ignored. It can be seen that LCP induces compressive residual stress on the surface layer of the specimens, and the compressive residual stress is higher without a coverage layer. The surface compressive residual stress values are −273 MPa and −252 MPa for the two cases, and the stress is significantly released from the top surface layer to a depth of 0.8 mm. The compressive residual stress at 0.8 mm specimen depth decreases to −24 MPa and −11 MPa. Then, the compressive residual stress decreases slowly with an increase in depth, before finally settling between −9 MPa and −11 MPa.

**Fig 15:**
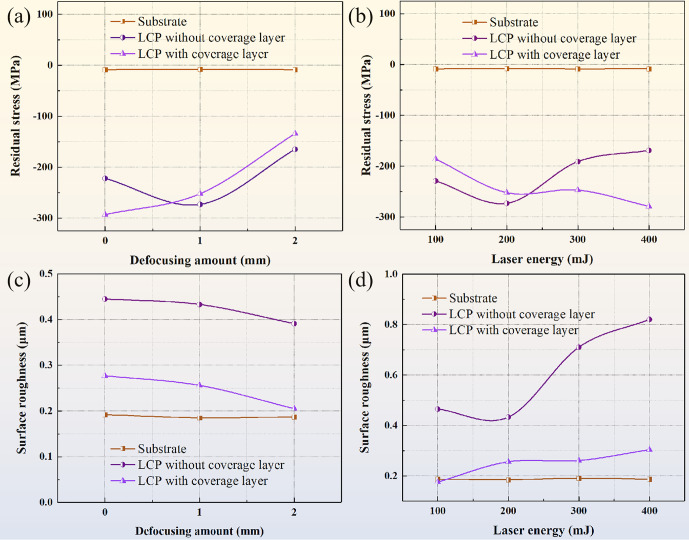
Residual stress and surface roughness (a) the residual stress under various defocusing amount, (b) the residual stress under various laser energies, (c) the surface roughness under various defocusing amount and (d) the surface roughness under various laser energies.

Fig 15(a) and 15(b) report the surface residual stress values of the LCP-treated specimens under various defocusing amounts and laser energies. As shown in Fig 15(a), under 0 mm defocusing amount, the residual stress for the no-coverage layer case is −221 MPa, while it is −293 MPa with a coverage layer. The residual stress decreases under a 2 mm defocusing amount due to the weaker influence of cavitation pulsation. It is worth noting that the maximum residual stress value in the case of no coverage layer occurs at −273 MPa at a 1 mm defocusing amount. The impact of the shock wave and water jet acts on the specimen surface directly, replacing the effect of laser ablation, which decreases due to the increase in defocusing amount. In Fig 15(b), with no coverage layer, the residual stress of the HT250 specimen increases first and then decreases with the increase in the laser energy; however, with a coverage layer, the residual stress increases with increasing laser energy. Compressive residual stresses improve materials’ mechanical properties; however, laser ablation may exert great influence on the material surface. The presence of a coverage layer may prevent the specimen surface from experiencing laser ablation damage; thus, the coverage layer is considered to protect the material surface from laser ablation.

Fig 15(c) and 15(d) show the surface roughness transformation trend of the LCP-treated specimens under various defocusing amounts and laser energies. The surface roughness increases after LCP treatment, and the roughness of the substrate is 0.19 μm. As shown in Fig 15(c), the surface roughness decreases with the increase in defocusing amount. For specimens without a coverage layer, the surface roughness values are 0.45 μm, 0.43 μm, and 0.39 μm, and for those without a coverage layer, the values are 0.28 μm, 0.26 μm, and 0.21 μm. As the defocusing amount increases, the shock wave and water jet impact weaken, reducing the probability of cavitation erosion. In addition, the presence of a coverage layer may result in a weaker LCP impact. As shown in Fig 15(d), a larger laser energy may result in an increase in roughness; however, for the no-coverage layer case, the roughness experiences a slight decrease initially before showing a significant increase. The increase in laser energy may cause gentle deformation of the material surface, resulting in a slight decrease in roughness. However, when the laser energy value reaches a high level, cavitation pits may appear on the material surface through the impact of the shock wave and water jet, leading to a sharp increase in surface roughness.

### Effect of coverage layer on electrochemical corrosion of cast iron after LCP treatment

In laser shock peening, a coverage layer is used as an absorption layer, which absorbs the laser energy and protects the material surface from laser damage. The absorption layer experiences explosive evaporation and generates high-pressure plasma, also producing a strong shock wave impacting the material surface [23,24]. LCP with laser shock peening is different, and impact-based LCP does not require the absorption of laser energy by a coverage layer. The laser incident in the liquid is used to induce a cavitation bubble, and the effect of LCP can be attributed mainly to the combined impact of the laser shock wave, water jet, and bubble collapse shock wave.

The effects of coverage layer presence on LCP under various laser energies and defocusing amounts are also very interesting. As the laser energy increases, the extent of laser breakdown liquid increases, and the bubble radius increases [25,26], so the laser shock wave is more intense at a higher laser energy. According to the Rayleigh formula based on cavitation bubble theory [27,28], the bubble energy is proportional to the cube of its maximum radius, so the bubble collapse shock wave and water jet are more intense at higher laser energy values. For the no-coverage layer case in this study, the impact of LCP was enhanced with the increase in laser energy at a certain range (100 mJ - 200 mJ), while the plastic deformation of the material surface became uneven as the laser energy was increased further (300 mJ - 400 mJ). Moreover, an increase in laser energy exacerbates the damage caused by cavitation erosion and laser ablation. The coverage layer prevents damage due to cavitation erosion and laser ablation, so the impact of LCP is always enhanced with an increase in the laser energy. Therefore, the extent of grain refinement and the induced residual stress are improved with increasing laser energy in the case of the coverage layer. The impact of the shock wave and water jet attenuate significantly with an increase in the defocusing amount. At 2 mm defocusing amount, the impact of LCP is very weak. In the no-coverage layer case, the focused laser beam is directly incident to the material surface under 0 mm defocusing amount and causes laser ablation, so the optimal parameter is 1 mm. With a coverage layer, the intensity of LCP is highest at 0 mm defocusing amount, and with this parameter, the material surface will not be ablated.

The electrochemical corrosion of HT250 gray cast iron in NaCl electrolyte solution can be considered a cyclic process characterized by the generation of an oxidation film and the invasion of Cl ions. Without a coverage layer, an increase in laser energy causes an obvious increase in surface roughness and the generation of cavitation erosion and laser ablation pits on the HT250 surface. The Cl ions are very small and are formed of highly soluble chloride interacting with metal ions, so they easily to adhere to the material’s uneven surface and destroy the integrity of the oxide film. Moreover, the potential electrode at the pit is usually more negative and can become an active anode, thus forming a clogged battery at the pit, and the corrosion is accelerated. Although the surface roughness is also increased after LCP treatment with a coverage layer, it is still low enough compared to LCP treatment without a coverage layer, and the HT250 surface will not have the corrosion pits. Therefore, the corrosion resistance of HT250 gray cast iron is better with the presence of a coverage layer.

Compared with laser shock peening (LSP), LCP is a relatively milder surface modification technology. Wang [29] used LSP to exert a residual compressive stress of 400 MPa on AISI 420 stainless steel, which numerically exceeds the 253 MPa reported in this study. The principles of the two methods are different: LSP mainly relies on the impact of plasma beneath a coverage layer, whereas LCP primarily utilizes the shock wave and water jet generated by cavitation bubble collapse. The coverage layer in this study can mitigate the side effects of laser ablation to a certain extent, rather than serving to guide the direction of the plasma shock as in laser shock peening. In addition, Even when using the same surface modification technology (LCP), different materials can influence the experimental results. Wang [30] applied the LCP method to Ti-30Zr-5Mo and induced a residual compressive stress of 179 MPa; Luo [31] treated a 1060 aluminum alloy with LCP using a different setup and induced a residual compressive stress of 50 MPa. As a result, the process details of LCP still require continuous optimization in the future.

## Conclusion

In this paper, we report on the electrochemical corrosion behavior of gray cast iron subjected to laser cavitation peening under different laser energies and defocusing amounts. With a coverage layer, the anti-corrosion ability of the experimental specimens increased with an increase in laser energy. The corrosion resistance of the specimens improved under all defocusing amounts after LCP treatment, and treatment at smaller defocusing amounts leads to higher corrosion resistance. The corrosion products are smaller and present as cotton-type products, while the number of cracks decreases significantly. Also, with a coverage layer, the corrosion resistance of gray cast iron first increases (0–200 mJ) with the increase in laser energy and then decreases (200 mJ - 400 mJ). The anti-corrosion ability after LCP treatment decreases significantly at 0 mm defocusing amount. The specimen surface has many lamellar and cotton-type corrosion products, and the distribution is uneven. The influence mechanism of the aluminum coverage layer was revealed, in combination with bubble pulsation evolution. The LCP process includes bubble evolution from laser breakdown to bubble expansion and from bubble contraction to collapse. The existence of the coverage layer prevents laser ablation and cavitation erosion. On the basis of material strengthening, the material surface is largely protected.

## References

[1] T. Faris S, Salih Mahdi H, N. Abed K. Studying and improving the hardness properties of gray cast iron. DJES. 2022:114–21. doi: 10.24237/djes.2022.15211

[2] Tonolini P, Montesano L, Pola A, Bontempi G, Gelfi M. Wear behavior of Nb alloyed gray cast iron for automotive brake disc application. Metals. 2023;13(2):365. doi: 10.3390/met13020365

[3] Ranjbar M, Razavi SH, Seyedraoufi ZS, Shajari Y. Investigating the effect of optimal addition of Inconel 718 machining swarfs on the wear behavior of gray cast iron. J Mater Res Technol. 2025;35:2558–72.

[4] Zhou YX, Zhang J, Xing ZG, Wang HD, Lv ZL. Microstructure and properties of NiCrBSi coating by plasma cladding on gray cast iron. Surf Coat Tech. 2019;361(1):270–9.

[5] Ahuir-Torres JI, Meredith A, Batako AD, Kotadia HR, Opoz TT, Zhu G, et al. Corrosion behaviour of hardened grey cast iron with continuous-wave infrared laser. Mater Today Commun. 2025;44:111852. doi: 10.1016/j.mtcomm.2025.111852

[6] Catalán N, Ramos-Moore E, Boccardo A, Celentano D. Surface laser treatment of cast irons: a review. Metals. 2022;12(4):562. doi: 10.3390/met12040562

[7] Chen ZK, Zhou T, Zhao RY, Zhang HF, Lu SC, Yang WS. Improved fatigue wear resistance of gray cast iron by localized laser carburizing. Mater Sci Eng A. 2015;644:1–9.

[8] Sai Q, Hao J, Wang S, Wang Z. Improving the properties of gray cast iron by laser surface modification. Materials (Basel). 2023;16(16):5533. doi: 10.3390/ma16165533 37629824 PMC10456106

[9] Park HI, Nakata K, Tomiada S. In situ formation of TiC particulate composite layer on cast iron by laser alloying of thermal sprayed titanium coating. J Mater Sci. 2000;35:747–55.

[10] Park IC, Lee HK, Kim SJ. Microstructure and cavitation damage characteristics of surface treated gray cast iron by plasma ion nitriding. Appl Surf Sci. 2019;477(477):147–53.

[11] Manoj A, Verma PC, Thangaraju S, Narala SKR, Saravanan P. High-temperature dry sliding tribological behavior of Fe-based laser cladded grey cast iron for automotive brake disc application. Wear. 2025;571:205819. doi: 10.1016/j.wear.2025.205819

[12] Wang J, Lu Y, Zhou D, Sun L, Xie L, Wang J. Mechanical properties and microstructural response of 2A14 aluminum alloy subjected to multiple laser shock peening impacts. Vacuum. 2019;165:193–8. doi: 10.1016/j.vacuum.2019.03.058

[13] Si C, Sun W, Tian Y, Cai J. Cavitation erosion resistance enhancement of the surface modified 2024T351 Al alloy by ultrasonic shot peening. Surf Coat Tech. 2023;452:129122.

[14] Soyama H, Simoncini M, Cabibbo M. Effect of cavitation peening on fatigue properties in friction stir welded aluminum alloy AA5754. Metals. 2020;11(1):59. doi: 10.3390/met11010059

[15] Soyama H, Kuji C. Improving effects of cavitation peening, using a pulsed laser or a cavitating jet, and shot peening on the fatigue properties of additively manufactured titanium alloy Ti6Al4V. Surf Coat Tech. 2022;451:129047.

[16] Sonde E, Chaise T, Boisson N, Nelias D. Modeling of cavitation peening: jet, bubble growth and collapse, micro-jet and residual stresses. J Mater Process Tech. 2018;262:479–91.

[17] Ijiri M, Shimonishi D, Tani S, Okada N, Yamamoto M, Nakagawa D, et al. Multifunction cavitation technology to improve the surface function of Al-Cu alloy. Int J Lightweight Mater Manuf. 2019;2:50–56.

[18] Ren XD, Wang J, Yuan SQ, Adu-Gyamfi S, Tong YQ, Zuo CY, et al. Mechanical effect of laser-induced cavitation bubble of 2A02 alloy. Opt Laser Technol. 2018;105:180–4. doi: 10.1016/j.optlastec.2018.02.039

[19] Ren XD, He H, Tong YQ, Ren YP, Yuan SQ, Liu R, et al. Experimental investigation on dynamic characteristics and strengthening mechanism of laser-induced cavitation bubbles. Ultrason Sonochem. 2016;32:218–23. doi: 10.1016/j.ultsonch.2016.03.012 27150764

[20] Zhang Z, Wei S, Wang P, Qiu W, Zhang G. Progress in applications of laser induced cavitation on surface processing. Opt Laser Technol. 2024;170:110212. doi: 10.1016/j.optlastec.2023.110212

[21] Trdan U, Grum J. SEM/EDS characterization of laser shock peening effect on localized corrosion of Al alloy in a near natural chloride environment. Corros Sci. 2014;82(1):328–38.

[22] Trdan U, Grum J. Evaluation of corrosion resistance of AA6082-T651 aluminium alloy after laser shock peening by means of cyclic polarization and ElS methods. Corros Sci. 2012;59:324–33.

[23] Lu JZ, Luo KY, Zhang YK, Sun GF, Gu YY, Zhou JZ, et al. Grain refinement mechanism of multiple laser shock processing impacts on ANSI 304 stainless steel. Acta Mater. 2010;58(16):5354–62. doi: 10.1016/j.actamat.2010.06.010

[24] Ren NF, Yang HM, Yuan SQ, Wang Y, Tang SX, Zheng LM, et al. High temperature mechanical properties and surface fatigue behavior improving of steel alloy via laser shock peening. Mater Des. 2014;53:452–6. doi: 10.1016/j.matdes.2013.07.009

[25] Zhang H, Ren X, Luo C, Tong Y, Larson EA, Lu Z, et al. Study on transient characteristics and influencing of temperature on cavitation bubbles in various environments. Optik. 2019;187:25–33. doi: 10.1016/j.ijleo.2019.01.076

[26] Li B, Zhang H, Lu J, Ni X. Experimental investigation of the effect of ambient pressure on laser-induced bubble dynamics. Opt Laser Technol. 2011;43(8):1499–503. doi: 10.1016/j.optlastec.2011.05.016

[27] Rayleigh L. On the pressure developed in a liquid during the collapse of a spherical cavity. Philos. Mag. Ser. 1917;634:94–8.

[28] Plesset M. The dynamics of cavitation bubbles. Appl Mech. 1949;16:227–90.

[29] Wang CY, Cheng W, Shao YK, Luo KY, Lu JZ. Cavitation erosion behaviour of AISI 420 stainless steel subjected to laser shock peening as a function of the coverage layer in distilled water and water-particle solutions. Wear. 2021;470–471:203611. doi: 10.1016/j.wear.2020.203611

[30] Wang R, Soyama H, Nakano T, Hosoda H, Niinomi M, Zhang X, et al. Enhancement of wear resistance in Ti–30Zr–5Mo implants via laser cavitation peening. Opt Laser Technol. 2026;201:115311. doi: 10.1016/j.optlastec.2026.115311

[31] Luo C, Gu J, Tong Z, Chen L, Zhou W, Wu K, et al. Dynamics of laser-induced cavitation bubbles near a short hole and laser cavitation processing with particles. Opt Laser Technol. 2021;135:106680. doi: 10.1016/j.optlastec.2020.106680

